# Implementation of a quality improvement project on smoking cessation reduces smoking in a high risk trauma patient population

**DOI:** 10.1186/s13017-016-0072-7

**Published:** 2016-04-26

**Authors:** Jeffry Nahmias, Andrew Doben, Shiva Poola, Samuel Korntner, Karen Carrens, Ronald Gross

**Affiliations:** Baystate Medical Center, affiliate of Tufts University School of Medicine, 759 Chestnut Street, Springfield, MA 01199 USA

**Keywords:** Trauma preventative care, Smoking, Smoking cessation

## Abstract

**Background:**

Cigarette smoking causes about one of every five deaths in the U.S. each year. In 2013 the prevalence of smoking in our institution’s trauma population was 26.7 %, well above the national adult average of 18.1 % according to the CDC website. As a quality improvement project we implemented a multimodality smoking cessation program in a high-risk trauma population.

**Methods:**

All smokers with independent mental capacity admitted to our level I trauma center from 6/1/2014 until 3/31/2015 were counseled by a physician on the benefits of smoking cessation. Those who wished to quit smoking were given further counseling by a pulmonary rehabilitation nurse and offered nicotine replacement therapy (e.g. nicotine patch). A planned 30 day or later follow-up was performed to ascertain the primary endpoint of the total number of patients who quit smoking, with a secondary endpoint of reduction in the frequency of smoking, defined as at least a half pack per day reduction from their pre-intervention state.

**Results:**

During the 9 month study period, 1066 trauma patients were admitted with 241 (22.6 %) identified as smokers. A total of 31 patients with a mean Injury Severity Score (ISS) of 14.2 (range 1–38), mean age of 47.6 (21–71) and mean years of smoking of 27.1 (2–55), wished to stop smoking. Seven of the 31 patients, (22.5 %, 95 % confidence interval [CI] of 10–41 %) achieved self-reported smoking cessation at or beyond 30 days post discharge. An additional eight patients (25.8 %, 95 % CI 12–45 %) reported significant reduction in smoking.

**Conclusions:**

Trauma patients represent a high risk smoking population. The implementation of a smoking cessation program led to a smoking cessation rate of 22.5 % and smoking reduction in 25.8 % of all identified smokers who participated in the program. This is a relatively simple, inexpensive intervention with potentially far reaching and beneficial long-term health implications. A larger, multi-center prospective study appears warranted.

LEVEL OF EVIDENCE: Therapeutic Study, Level V evidence.

## Background

According to the World Health Organization (WHO), in 2012, 21 % of the global population smoked tobacco [1]. Annually, tobacco usage worldwide causes the direct death of over 5 million people with an additional 600,000 people dying from second-hand smoke [2]. In the United States alone, cigarette smoking accounts for one of every five deaths each year [3]. As compared to nonsmokers, the life expectancy of smokers is lowered by 13.2 years for men and 14.5 years for women [4]. Smokers are at an increased risk for chronic obstructive pulmonary disease (COPD) with an absolute risk (AR) of 25 % and lung cancer with an average relative risk (RR) of 15 to 30 [5, 6]. Smoking also increases the likelihood of developing chronic diseases, such as ischemic heart disease (RR = 2.2), cerebrovascular disease (RR = 1.6) and atherosclerotic cardiovascular disease (RR = 1.6) [7]. Smoking is linked to countless malignancies including 40–70 % of bladder cancers and 30 % of pancreatic cancers [8].

Additionally, cigarette smoking poses a financial burden. Assuming a pack of cigarettes costs 7 dollars, smoking one pack a day will cost an individual over $2500 per year. Nationally, from 2001 to 2004, the average financial burden of smoking was approximately $193 billion per year. Of this amount, $96 billion is attributed to health care cost, while the remaining $97 billion is accounted from lost productivity [9].

The Center for Disease Control (CDC) estimates that there are over 42 million adults in the United States who smoke cigarettes [10]. In 2010, the CDC reported that approximately 69 % of smokers were interested in quitting and 52 % of smokers had made a quit attempt in the past year [11]. Multimodal interventions, specifically combined nicotine replacement therapy (NRT) and counseling have demonstrated cessation rates from 20–33 % [12].

A national survey documented an overall decline of cigarette smoking from 21 % in 2005 to 18 % in 2013 [10]. In 2013, only 16.6 % of adults were reported to be smokers in the state of Massachusetts [13]. However at our institution in 2013, the prevalence of smoking among our trauma population was much higher at 27 %. The American College of Surgeons (ACS) has tasked all trauma centers with providing education on alcohol cessation, as studies have shown alcohol to be linked to traumatic injuries. Unlike this stance on alcohol, the ACS has no mandate for smoking cessation counseling. Prior to implementation of this program, our institution had no formalized protocol for smoking cessation. Previous studies have demonstrated that impulsivity is related to heightened nicotine dependence; however the exact relationship is far more complex than a simple causal relationship [14]. The purpose of this study was to implement a multimodality smoking cessation program in a high-risk trauma population. We hypothesized this would lead to smoking cessation in our trauma patient population who previously received no care to address smoking.

## Methods

After obtaining Institutional Review Board exemption, all smokers with independent mental capacity were enrolled from June 1, 2014 to March 31^st^, 2015 at Baystate Medical Center, a level one trauma center. Participants received initial physician counseling regarding smoking cessation. All physician counselors were given a document compiled by the investigators before the start of the intervention. This included detrimental effects of smoking, benefits of smoking cessation, and responses for common reasons that patients refuse to stop smoking. If the patient desired smoking cessation, demographics including: gender, age, injury severity score (ISS), thorax abbreviated injury scale (AIS), and number of years smoking were obtained. They also were given educational pamphlets regarding benefits of smoking cessation and outpatient resources. All patients were offered nicotine replacement therapy (NRT) via a nicotine patch.

If admitted, a pulmonary rehabilitation nurse consultation with further counseling was ordered. Patients agreed to participate in an outpatient survey at 30 days or later. This survey assessed the primary endpoint of smoking cessation as well as the secondary endpoint of significant reduction in smoking defined as at least half a pack per day.

Descriptive statistics (mean and range) for continuous variables and percents for categorical variables were reported for all demographic and clinical variables. Rates of successful smoking cessation and significant smoking reduction and their 95 % confidence intervals were calculated.

## Results

During the 9 month study period from June 1^st^, 2014 until March 31^st^, 2015, 1066 patients were admitted to Baystate Medical Center, a level one trauma center. Two-hundred forty one patients (22.6 %) were identified as smokers. Only 31 patients (23 male, eight female) wished to pursue smoking cessation. These patients had a mean ISS of 14.2 (range 1–38) and mean thorax AIS of 2.1 (0–5). The participants had a mean age of 47.6 (21–71) and mean years of smoking of 27.1 (2–55). A 100 % survey response was achieved at the 30 day or later designated follow-up. All participants were outpatients at this point with the capability to re-start smoking if desired. Seven of the 31 patients, (22.5 %, 95 % Confidence Interval Confidence Interval [CI] of 10–41 %) achieved smoking cessation at the follow-up. Eight patients (25.8 %, 95 % [CI] of 12–45 %) reported significant reduction in smoking, defined by at least half a pack per day reduction (Table 1). Thirteen of the 24 patients who did not achieve smoking cessation still desired to quit. The most common impediment to smoking cessation of those unable to quit was withdrawal symptoms (20.8 %). The second most common impediment was an inability to procure nicotine replacement therapy as an outpatient (16.7 %). An additional 4.1 % of remaining smokers cited alcohol influence and lack of motivation. On univariate analysis, comparing the subgroups of those who achieved 30 day cessation and those who did not, three variables were statistically significant (*p* < .05): LOS, ISS, and Thorax AIS (Table 2). After controlling for ISS in multiple logistic regression, the difference between the smoking cessation group and non-cessation group in LOS was not statistically significant (*p* = 0.19).

**Table 1:** Description of participants

Participants	31
Male	23
Female	8
Mean age (Range)	47.6 (21–71)
Mean ISS (Range)	14.2 (1–38)
Mean years smoking (Range)	27.1 (2–55)
Stopped smoking at 30 days (percent)	7/31 (23 %)
Significantly decreased smoking (percent)	8/31 (26 %)

**Table 2:** Demographic and outcomes analysis

Variable	No (Mean/CI)	Yes (Mean/CI)	Significance
Age	46.9(41.6–52.3)	50(33.5–66.5)	0.61
Years smoked	26.3(20.7–32.0)	30(10.0–50.0)	0.58
LOS(CI)	4.3 (2.8–5.8)	7.6 (4.6–10.6)	0.0333
ISS(CI)	11.4 (7.8–15.1)	21.1 (13.5–28.8)	0.0133
Thorax AIS(CI)	1.6 (1.0–2.3)	3.6 (2.7–4.5)	0.0031
Readmit rate	8.30 %	14.30 %	0.639

## Discussion

Smoking is associated with harmful effects on every organ in the body and is well recognized as the leading cause of preventable illness and death [15]. Smoking adversely affects orthopedic injuries. A recent systematic review was performed by Scolaro et al. included 19 papers (seven prospective, 12 retrospective cohort) examining the effects of smoking on orthopedic trauma. They discovered an adjusted odds ratio of nonunion in smokers of 2.32 and an approximately 6 week longer mean healing time compared to nonsmokers. There was also a trend toward more postoperative wound infections [16].

The link between smoking and pulmonary disease is even more established. The pathophysiology of this process is beyond the scope of this manuscript, but some mechanisms include direct epithelial injury, carcinogen exposure, oxidative stress and mucociliary dysfunction [17]. Smoking is shown to have deleterious effects on respiratory failure in trauma. A retrospective review by Resnick et al. found smokers on average spent five more days of mechanical ventilation compared to nonsmokers [18]. Smoking is also considered one of the main modifiable risk factors for all types of pneumonia, including ventilator-associated pneumonia [19]. Calfee et al found smoking to be a risk factor for acute respiratory distress syndrome (odds ratio 2.28) in patients even with non-pulmonary sepsis [20]. In a prospective observational study conducted by Lucidarme on mechanically ventilated patients, there was a significant difference in morbidity, especially agitation, self-removal of tubes and need for sedatives and restraints when comparing smokers versus nonsmokers [21].

Smoking has also been associated with impaired wound healing. Smoking has deleterious effects on neutrophil responsiveness and migratory and bactericidal function. Smoking also decreases mucosal and subcutaneous blood flow by as much as 40 % in some studies. It additionally causes oxidative stress through creation of reactive oxygen species [22].

In 2006, the American College of Surgeons Committee on Trauma (COT) published their first document on Alcohol Screening and Brief Intervention for Trauma Patients [23]. The need for this document is obvious given that nearly 50 % of trauma admissions have a positive blood alcohol level. In our trauma population we found a significant rate of smoking, 22.6 %. A retrospective chart review by Ferro et al. found an even higher smoking rate of 42.9 % in their trauma population [24]. Hence, like alcohol, this is a common problem in the injured patient.

Smoking cessation creates immediate benefits, which help the injured patient. Within hours of cessation, tissue blood flow and oxygen levels return to normal. At approximately two weeks of abstinence platelet function normalizes. Vitamin C levels and collagen synthesis appear to take approximately 4 weeks to normalize, although some benefit is seen earlier. By 6–8 weeks mucociliary function normalizes and perioperative complications decline. The effects of nicotine replacement therapy is not clear, a systematic review by Sorensen concluded that there is no evidence to suggest that nicotine replacement therapy has a detrimental or beneficial effect on postoperative outcomes or wound healing [22].

In terms of our patient population, there was no difference between those who achieved cessation and those who did not in terms of age, years of smoking, or readmission rate. There was a statistically significant difference in Thorax AIS, ISS, and LOS. Those achieving smoking cessation had a higher thorax AIS. It makes sense that patients with significant pulmonary injuries would be more amenable to cessation as they can easily comprehend the cause and effect relationship. ISS in the smoking cessation group was significantly higher however we do not believe there is a causal relationship. Finally, in terms of LOS being significantly higher for cessation patients, as previously mentioned this difference was no longer statistically significant when we controlled for ISS. Larger studies will be required to better elucidate which patients to focus on for counseling and what potential short term benefits can be achieved with smoking cessation in trauma patients.

The long term benefits of smoking cessation are quite profound. According to the WHO and CDC at one year from cessation the risk of coronary artery disease is half of a smokers and by 5 years the risk of stroke is that of a nonsmoker [1, 2, 4].

The importance of smoking cessation is clear for all patients, but should have the utmost significance for those who are traumatically injured. In our study, we achieved a smoking cessation rate of 22.5 %. The literature contains varied success rates depending on the modalities utilized, patient population and endpoint time, as there is certainly a component of recidivism. A Cochrane review of physician counseling for smoking cessation included 42 trials and found a 1–3 % improvement in cessation with only physician counseling, however, noted a higher rate with more intensive counseling and/or follow-up [25]. A recent randomized trial by Halpern et al. found a 30 day abstinence rate ranging from 10.5 to 22.6 % using financial incentives [26]. Other studies that utilize a multimodality approach, including behavioral therapy, counseling, nicotine replacement and pharmacologic therapy, have reported success rates ranging from 15–68 % [11, 12, 27]. Eisenberg et al. performed a meta-analysis, which included only randomized controlled trials, found an abstinence rate of 16.4 % for nicotine replacement therapy alone, 30.3 % for pharmacologic therapy alone and 35.5 % for combination therapy [28]. Other studies that include nicotine replacement therapy plus behavioral counseling have found abstinence rates ranging from 20–33 % depending on the intensity of counseling [12].

When assessing our financial and fixed resources, our smoking cessation program would need to be easy to implement and cost effective. Thus, the upfront cost of financial incentives was deemed prohibitive. In terms of pharmacologic therapy there are 2 FDA approved options, Varenicline (Chantix) and Buproprion (Zyban). Buproprion therapy is associated with an alarmingly high rate of insomnia (30–50 %), dry mouth (11 %) and other less common side effects such as tremor (3.4 %), rash (2.4 %), and seizures (0.1 %) [29]. Varenicline has two major safety concerns, namely neuropsychiatric and cardiovascular. The latter led to an FDA advisory in 2011 that there may be increased risk of cardiovascular events in patients with known cardiovascular disease. Other commonly reported side effects include insomnia, nausea, visual disturbances, syncope and moderate to severe skin reactions. Given the relative frequency of adverse medication reactions, we felt it prudent to only use nicotine replacement therapy despite a slightly inferior cessation rate. In the future, we realize that engagement of our primary care physicians to assist with the prescription and maintenance of these medications may be helpful as the most common impediment to quitting was withdrawal symptoms, which may be ameliorated via these medications.

Furthermore, U.S. insurance plans are required to cover tobacco-cessation interventions including behavioral counseling and medications approved by the Food and Drug Administration. This may alleviate our second most common cited impediment to quitting, which was an inability to procure nicotine replacement therapy.

In addition to our rate of cessation we also achieved a 25.8 % reduction in smoking, which we defined by half a pack per day. Multiple studies have shown that even in those not interested in quitting, a reduction in smoking leads to a higher future rate of smoking cessation [30].

Some of the limitations to our study include lack of power, non-randomization, and length of follow-up. In terms of sample size, this was originally designed as a quality improvement project that achieved good results, we hope to continue this process of accrual and potentially expand it to our emergency general surgery patients as well. Since this is not a randomized controlled-blinded trial there are inherent biases such as selection bias as we did not control for potential confounders, including recent attempts to quit, concurrent substance abuse or other factors that may be unrecognized impediments. Additionally, with any interview there is a potential for interviewer bias; an attempt to minimize this was achieved by using a standardized script questionnaire. Finally, there is certainly a known potential for response bias, namely false positives for smoking cessation. Although in the setting of a non-financial gain scenario (i.e. no financial incentive for quitting), this is minimized as much as possible short of confirmatory nicotine testing.

## Conclusion

Smoking has significant financial and health ramifications for trauma patients and healthcare as a whole. The implementation of a smoking cessation program led to a smoking cessation rate of 22.5 % and smoking reduction in 25.8 % of all identified smokers who participated in the program. This is a relatively simple and inexpensive intervention with potentially far reaching and beneficial long-term health implications. A multicenter evaluation including trauma and potentially emergency general surgical patients is warranted.

### Ethics approval and consent to participate

The authors assert that all procedures contributing to this work comply with the ethical standards of the relevant national and institutional committees on human experimentation and with the Declaration of Helsinki.

The authors assert that this study was deemed exempt from the institutional review board of Baystate Medical Center.

### Consent for publication

The authors assert this manuscript does not contain any individual person’s data in any form.
